# Phase 1b Study of Tovorafenib, Plozalizumab or Vedolizumab Plus Standard-of-Care Immune Checkpoint Inhibitors in Patients with Advanced Melanoma

**DOI:** 10.7150/jca.117878

**Published:** 2025-08-11

**Authors:** Ryan J. Sullivan, Katy K. Tsai, Anna C. Pavlick, Elizabeth I. Buchbinder, Sanjiv S. Agarwala, Antoni Ribas, Johan Jansson, Guillermo Rossiter, Anthony J. Olszanski

**Affiliations:** 1 Cancer Center, Massachusetts General Hospital, Boston, MA, USA.; 2 Helen Diller Family Comprehensive Cancer Center, University of California, San Francisco, San Francisco, CA, USA.; 3 Weill Cornell Medicine-Meyer Cancer Center, New York, NY, USA.; 4 Department of Medical Oncology, Dana-Farber Cancer Institute, Boston, MA, USA; 5 Lewis Katz School of Medicine at Temple University, Philadelphia, PA, USA.; 6 Jonsson Comprehensive Cancer Center at the University of California, Los Angeles, Los Angeles, CA, USA.; 7 Takeda, Cambridge, MA, USA.; 8 Fox Chase Cancer Center, Philadelphia, PA, USA.

**Keywords:** Checkpoint blockade, Combination immunotherapy, Melanoma, Plozalizumab, Tovorafenib, Toxicities, Vedolizumab

## Abstract

**Background:** Novel advanced melanoma therapy combinations may increase treatment efficacy and reduce treatment-related toxicities.

**Methods:** This open-label, nonrandomized, multicenter, phase 1b, 3-arm, umbrella study enrolled patients with advanced melanoma eligible for standard-of-care checkpoint inhibitor therapy. There were 3 phases: dose escalation; Part 1 limited cohort expansion; Part 2 additional expansion. Arms (A) 1, 2, and 3 investigated tovorafenib plus nivolumab, plozalizumab plus nivolumab, and vedolizumab plus nivolumab plus ipilimumab, respectively. In the dose-escalation plus Part 1 limited cohort expansion phase, the primary endpoint was dose-limiting toxicities.

**Results:** Twenty-two patients (A1=1; A2=9; A3=12) were enrolled before premature study termination. A1 was closed due to lack of enrollment. A2 enrollment was closed due to lack of clinical benefit (6/9 patients discontinued due to disease progression), and A3 enrollment was closed due to meeting prespecified stopping criteria (grade 3 diarrhea/colitis in 2 patients). One patient (A2) experienced dose-limiting toxicities. Grade ≥3 adverse events were reported in the single patient from A1, 3 (33.3%) patients from A2, and 10 (83.3%) patients from A3.

**Conclusion:** Study design allowed early termination after initial results suggested unlikely clinical benefit. Efficacy remains inconclusive for tovorafenib plus nivolumab and vedolizumab plus nivolumab plus ipilimumab in advanced melanoma. Trend review in this small population suggests a limited effect of investigated vedolizumab regimens as primary prophylaxis against nivolumab plus ipilimumab gastrointestinal toxicity.

## Introduction

Standard-of-care (SOC) treatment options for patients with advanced (unresectable and/or metastatic) melanoma include immunotherapy with checkpoint inhibitors (nivolumab, pembrolizumab, nivolumab plus ipilimumab followed by nivolumab, or nivolumab plus relatlimab) 1. Nivolumab, a programmed cell death protein-1 inhibitor (anti-PD-1), and ipilimumab, a cytotoxic lymphocyte antigen-4 inhibitor (anti-CTLA-4), are approved for use as single agents and as a dual checkpoint inhibitor combination therapy for patients with unresectable or metastatic melanoma 2, 3. Although the nivolumab plus ipilimumab combination is associated with >50% response rate and a survival benefit versus nivolumab alone or ipilimumab alone, >50% of patients treated with nivolumab plus ipilimumab experience treatment-related grade 3/4 toxicity, which may lead to treatment discontinuation 4-7. Colitis and diarrhea are among the leading causes of treatment discontinuation for this combination therapy 7.

Standard treatment options in patients who have advanced melanoma with rapidly accelerated fibrosarcoma (RAF) B-type proto-oncogene (*BRAF*) V600 mutations include immune checkpoint inhibition described previously as well as combinations of targeted therapies with BRAF and mitogen-activated extracellular signal-regulated kinase (MEK) inhibitors: dabrafenib plus trametinib, encorafenib plus binimetinib, and vemurafenib plus cobimetinib 1. BRAF and MEK inhibitor combination therapies have shown response rates of approximately 60%-70%; however, grade 3/4 adverse events (AEs) were also reported in >50% of patients receiving these combination treatments 8-10. Furthermore, response to combined BRAF and MEK inhibitor therapy is often transient due to development of resistance, leading to disease relapse 11.

The unmet need to improve treatment tolerability, response rate, durability of response, and survival persists despite the advances in melanoma treatment 12. Combinations of BRAF and MEK inhibitors with immunotherapy have been evaluated in randomized phase 2 and 3 trials suggesting potential but limited benefits in progression-free survival with triple therapy (BRAF and MEK inhibitors plus immunotherapy) versus double therapy (BRAF and MEK inhibitors) 13-15. Indeed, one such regimen, combined vemurafenib, cobimetinib, and the anti-PD-L1 atezolizumab received FDA-approval 14. However, a higher rate of grade 3/4 AEs with triple versus double therapy has also been reported 13-15.

Tovorafenib (previously known as MLN2480, TAK-580, and DAY101) is an oral, selective, central nervous system-penetrant, small-molecule, type II pan-RAF inhibitor; tovorafenib is active against both monomeric and dimeric forms of RAF signaling 16. Tovorafenib monotherapy has been investigated in a phase 1 study of patients with advanced solid tumors, which also included a dose-expansion phase in patients with metastatic melanoma (NCT01425008) 17. Based on the interim results of an ongoing phase 2 study of tovorafenib in pediatric, adolescent, and adult patients with recurrent or progressive low-grade glioma or an advanced solid tumor harboring a known *RAF* alteration (FIREFLY-1; NCT04775485) 18, tovorafenib monotherapy was approved in the United States for the treatment of patients aged ≥6 months who had relapsed or refractory pediatric low-grade glioma harboring a *BRAF* fusion, *BRAF* rearrangement, or *BRAF* V600 mutation 19. A phase 1b/2 study of tovorafenib monotherapy and combination of tovorafenib plus MEK inhibitor pimasertib in patients with recurrent, progressive, or refractory melanoma or other solid tumors with alterations in the key proteins of the mitogen-activated protein kinase (MAPK) pathway (FIRELIGHT; NCT04985604) 20 and a phase 3 study of tovorafenib monotherapy versus SOC chemotherapy in patients with pediatric low-grade glioma harboring an activating *RAF* alteration who require first-line systemic therapy (LOGGIC/FIREFLY-2; NCT05566795) are ongoing 21. Because a combination of BRAF and MEK inhibitors with immunotherapy has been shown to increase cytotoxic T-cell infiltration in the tumor and enhance antitumor activity versus immunotherapy alone in a mouse melanoma model as well as in patients treated with vemurafenib plus atezolizumab with or without cobimetinib 22, 23, we hypothesized that tovorafenib in combination with nivolumab may enhance cytotoxic T-cell infiltration, leading to improved clinical efficacy versus nivolumab alone. Another open-label phase 2 study is investigating a combination of tovorafenib plus nivolumab in children and young adults with craniopharyngioma 24.

Plozalizumab (previously known as TAK-202) is a genetically engineered, humanized monoclonal antibody of the immunoglobulin class that is a potent specific antagonist of cysteine-cysteine chemokine receptor type 2 (CCR2). Myeloid-derived suppressor cells overexpress CCR2 and are preferentially recruited to the tumor microenvironment, where they support tumor cell dissemination and inhibit T-cell function 25-27. We hypothesized that plozalizumab in combination with checkpoint inhibition has the potential to block circulating myeloid-derived suppressor cells that overexpress CCR2 from trafficking to the tumor microenvironment.

Vedolizumab is a gut-selective, recombinant, humanized monoclonal antibody that binds specifically to the human lymphocyte integrin α_4_β_7_ and acts as an immunomodulator 28. Vedolizumab is approved for the treatment of inflammatory bowel disease (IBD), a condition characterized by inflammation of the gastrointestinal tract 29. Several case series and retrospective analyses reported that vedolizumab was associated with a reduction of immune-related colitis in patients with cancer treated with immune checkpoint inhibitors 30-32. We hypothesized that the administration of prophylactic vedolizumab to patients who are receiving nivolumab plus ipilimumab combination therapy could prevent or ameliorate gastrointestinal immune-mediated AEs, thereby improving the safety profile of combination checkpoint inhibitor therapy. Notably, at the time of study launch, this approach had not been well described and subsequent trials adding the IL-6 receptor antagonists tocilizumab and sarilumab to frontline immunotherapy for patients with metastatic melanoma (NCT03999749, NCT04940299, NCT05428007) had not been started or reported 33-35.

Here, we report the results from a phase 1b study that investigated tovorafenib plus nivolumab, plozalizumab plus nivolumab, and vedolizumab plus nivolumab plus ipilimumab in patients with advanced melanoma.

## Methods

### Study design

This was an open-label, multicenter, phase 1b, 3-arm, umbrella study (NCT02723006, EudraCT 2015-005554-35) in patients with advanced/metastatic melanoma who were eligible to receive either anti-PD-1 or anti-PD-1 plus anti-CTLA-4 SOC therapy. The study was conducted in accordance with the International Council for Harmonisation Harmonised Tripartite Guideline for Good Clinical Practice and the ethical principles described in the Declaration of Helsinki, as well as applicable local or regional regulatory requirements. Institutional review board/independent ethics committee approval was obtained for all participating sites.

The study was not randomized. Following screening, patients were assigned by the investigator to 1 of 3 treatment arms according to either the medical characteristics of the patient that may have favored enrollment in a specific arm (i.e., previous treatments, *BRAF* mutation status, suitability for nivolumab plus ipilimumab combination) or enrollment efficiency to complete cohorts during dose escalation.

**Figure 1:**
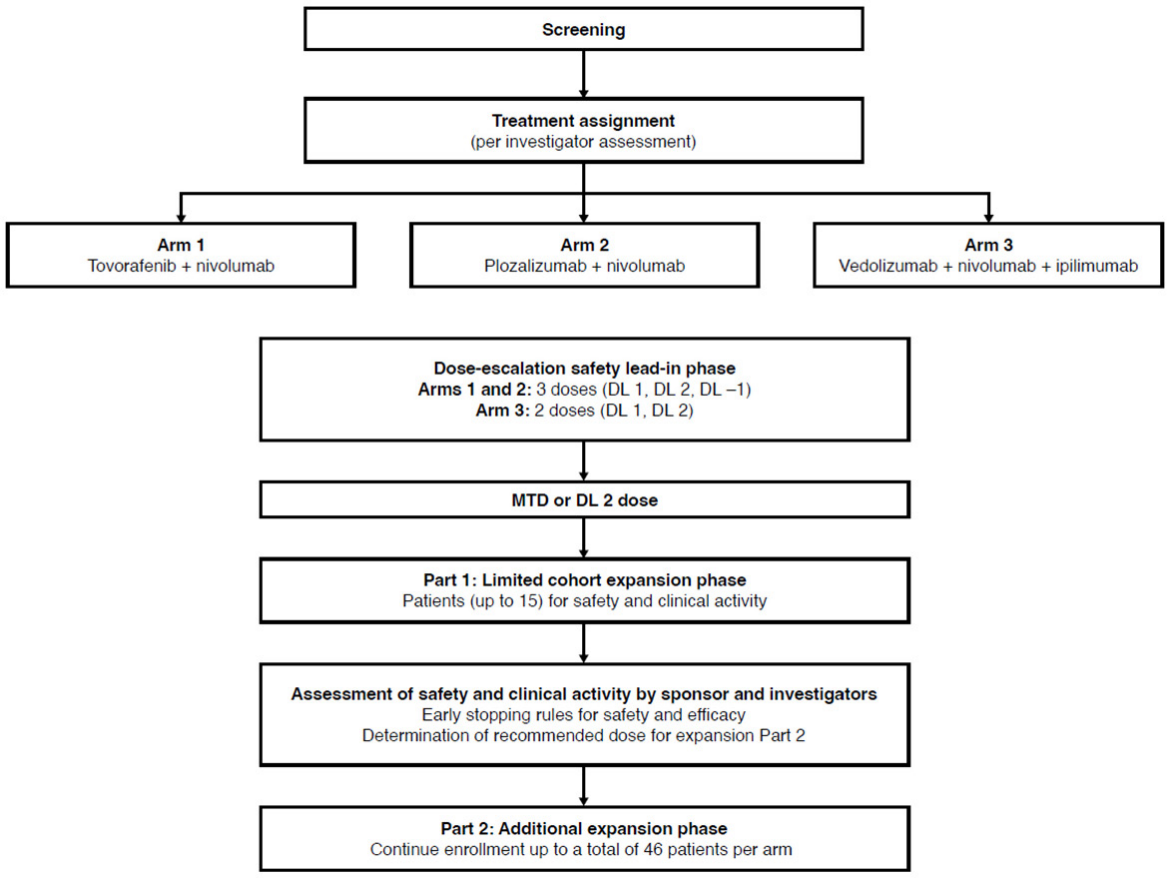
Study design. *DL,* dose level; *MTD,* maximum tolerated dose.

The drug combinations used in the treatment arms are summarized in **Figure 1**. Patients in Arm 1 received tovorafenib plus SOC nivolumab. Tovorafenib dose levels (DLs) were 300 mg, 400 mg, and 600 mg for DL -1, DL 1, and DL 2, respectively, and the drug was administered orally once weekly starting at week 1, day 1. Nivolumab was administered intravenously at 3 mg/kg (or 240 mg flat dose) every 2 weeks starting at week 3, and at least 1 hour after oral administration of tovorafenib.

Patients in Arm 2 received plozalizumab plus SOC nivolumab. Plozalizumab DLs were 2 mg/kg, 4 mg/kg, and 8 mg/kg for DL -1, DL 1, and DL 2, respectively, and the drug was administered intravenously at week 1, day 1; week 3, day 15; week 5, day 29; and every 4 weeks thereafter. Nivolumab was administered intravenously at 3 mg/kg (or 240 mg flat dose) every 2 weeks starting at week 3, and at least 30 minutes after plozalizumab.

Patients in Arm 3 received vedolizumab plus SOC combination of nivolumab plus ipilimumab. Vedolizumab DLs were 200 mg for DL 1 and 450 mg for DL 2, and the drug was administered intravenously at week 1, day 1; week 3, day 15; week 5, day 29; and week 13, day 85. Nivolumab was administered intravenously at 1 mg/kg every 3 weeks, starting at week 1 for 4 doses, and then intravenously at 3 mg/kg (or 240 mg flat dose) every 2 weeks starting at week 13. Ipilimumab was administered at 3 mg/kg intravenously every 3 weeks, starting at week 1 for 4 doses. At week 1, day 1, vedolizumab was administered first, followed by nivolumab and ipilimumab, with at least 30 minutes between treatments.

The study consisted of 3 phases to be conducted over a maximum 50-week treatment period: dose-escalation safety lead-in phase, Part 1 limited cohort expansion (expansion Part 1: a limited cohort expansion confirmatory safety phase), and Part 2 additional expansion (expansion Part 2: an additional cohort expansion phase) (**Figure 1**). The dose-escalation phase followed 3+3 escalation rules, starting with the treatment of a cohort of 3 patients at planned DL 1 of tovorafenib, plozalizumab, or vedolizumab. The rules for dose escalation to DL 2 or de-escalation to DL -1 (in Arms 1 and 2 only) to determine the dose for expansion Part 1 (defined as the maximum tolerated dose) are shown in **Figure S1**. For each cohort of 3 patients, dose-limiting toxicities (DLTs) were evaluated through the first 8 weeks of treatment for Arms 1 and 2, and for the first 6 weeks of treatment for Arm 3 before enrollment of the next cohort of 3 patients.

No formal interim analysis was planned for this study; a thorough safety and efficacy evaluation was to be performed before entering expansion Part 2. Termination of each treatment arm independently from other treatment arms was allowed.

The dose-escalation safety lead-in phase was the only phase of the study started before the study was terminated prematurely. The remaining phases of this study are not described in detail; however, the overall study design is depicted in **Figure 1**.

### Study population

Adult male or female patients (aged ≥18 years) with histologically confirmed unresectable stage III or stage IV melanoma per the American Joint Committee on Cancer (AJCC) staging system and with an Eastern Cooperative Oncology Group (ECOG) performance status of 0-1 were eligible to participate in the study. Patients had to be eligible for treatment with nivolumab or nivolumab plus ipilimumab at the dose(s) and schedule(s) recommended as SOC. For Arm 1, only patients with *BRAF* V600 mutation-positive or neuroblastoma rat sarcoma viral oncogene homolog (*NRAS*) mutation-positive disease previously untreated with RAF, MEK, or other inhibitors of the MAPK pathway were included. Written informed consent was obtained from all patients. Full inclusion and exclusion criteria are provided in **Appendix 1**.

### Study endpoints and assessments

#### Primary endpoint

The primary endpoint in the dose-escalation phase plus the expansion Part 1 was the frequency of DLTs. This primary endpoint would be used to define the recommended dose for the expansion Part 2, with secondary safety endpoints being considered for the final dose determination.

#### Secondary safety endpoints

The secondary safety endpoints to define the recommended dose for the expansion Part 2 included frequency and severity of treatment-emergent AEs (TEAEs) per National Cancer Institute Common Terminology Criteria version 4.03, serious TEAEs, treatment discontinuation rates, and dose modifications.

#### DLTs and stopping rules

DLTs were any of the following events that were considered by the investigator to be at least possibly related to study treatment (tovorafenib, plozalizumab, and/or vedolizumab either as a single agent or in combination with nivolumab or nivolumab plus ipilimumab): delay in the administration of the scheduled treatment of ≥3 weeks due to a lack of adequate recovery from treatment-related toxicity (recovery to grade 1 or lower, or to the patient's baseline, or to a level considered acceptable by the investigator); other treatment-related grade 2 nonhematologic toxicities that, in the opinion of the investigator, should also be considered as dose limiting; and any grade 3 or higher AE that is assessed as at least possibly related to study drug.

Hematologic toxicities that were not considered DLTs included grade 4 neutropenia (absolute neutrophil count < 500 cells/mm^3^) lasting < 7 days in the absence of fever > 38.5 °C sustained for > 1 hour; grade 3 neutropenia of any duration in the absence of fever > 38.5 °C sustained for > 1 hour; grade 3 anemia in patients with history of transfusion supportive care or in patients participating in Arm 1 (tovorafenib plus nivolumab); and grade 3 thrombocytopenia without bleeding.

Nonhematologic toxicities that were not considered DLTs included nausea and vomiting persisting at grade 3 for ≤ 3 days after instituting supportive care measures including oral/intravenous antiemetic medications; isolated grade 3 or higher laboratory abnormalities if asymptomatic and resolve to grade 2 or lower or baseline levels in ≤ 7 days; grade 3 asymptomatic hypophosphatemia (Arm 1 only); grade 3 arthralgia/myalgia that responds to nonsteroidal anti-inflammatory drug use; grade 3 fatigue; and grade 3 rash lasting ≤ 7 days after treatment that includes topical steroids, oral antihistamines, and pulse oral steroids (if necessary).

For Arm 3, if 2 or more patients presented with grade 3 or higher diarrhea or colitis and/or more than 7 patients presented with diarrhea/colitis of any grade, enrollment to expansion Part 2 was not to be initiated.

#### Early study termination

Due to early termination of the study in the dose-escalation phase, this report describes patient disposition and TEAEs for each of the 3 treatment arms. A formal analysis of efficacy data was not performed, and treatment response using Response Evaluation Criteria in Solid Tumors, version 1.1 (RECIST v1.1) guidelines was evaluated only descriptively.

### Statistical analyses

In this study, the described analysis set comprised the safety population, defined as all patients who received at least 1 dose, even if incomplete, of study treatment (tovorafenib, plozalizumab, or vedolizumab). Data by treatment arm are presented using a descriptive statistical analysis, with mean, SD, median, and range for continuous variables and the number and percentage per category for categorical data.

### Data availability

The datasets, including the redacted study protocol, redacted statistical analysis plan and individual participants' data supporting the results reported in this article will be made available within 3 months from initial request to researchers who provide a methodologically sound proposal. The data will be provided after its de-identification, in compliance with applicable privacy laws, data protection, and requirements for consent and anonymization.

## Results

### Study population and patient disposition

In total, 22 patients were enrolled in the study from centers in the United States and comprised the safety population analysis set from 2016 - 2018. Study enrollment started in June 2016 and the study was terminated prematurely in May 2018. One patient was enrolled in Arm 1 (tovorafenib plus nivolumab), 9 in Arm 2 (plozalizumab plus nivolumab), and 12 in Arm 3 (vedolizumab plus nivolumab plus ipilimumab). Patient demographic and baseline characteristics are show in **Table 1**. Study population representativeness is described in **Table S1**.

**Figure 2:**
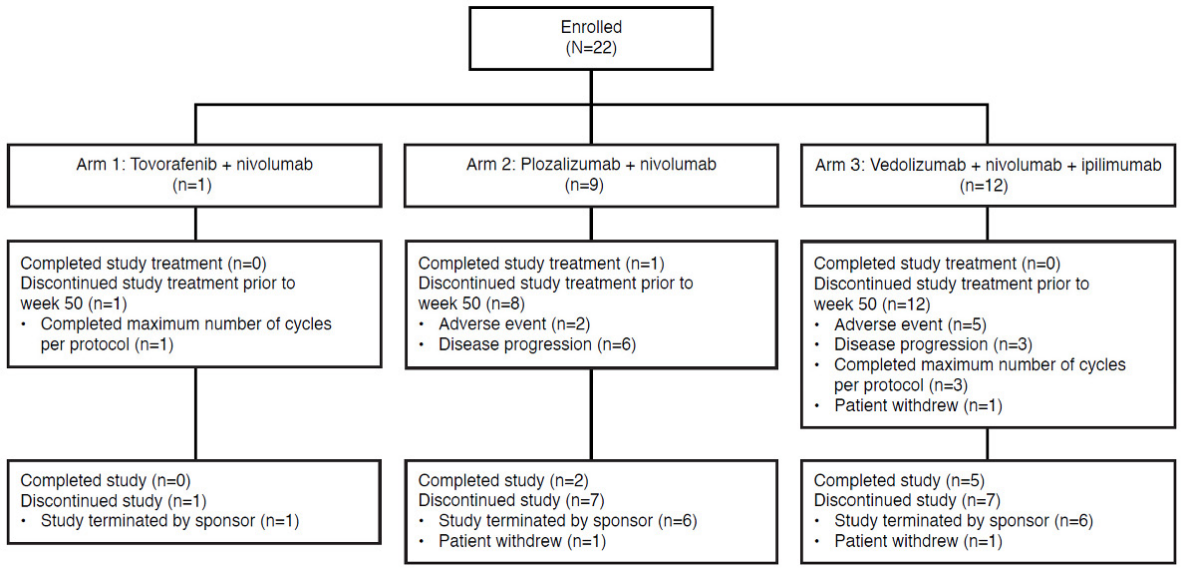
Patient disposition.

Seven of 22 patients (31.8%) completed the study: 2 of 9 patients (22.2%) from Arm 2 (plozalizumab plus nivolumab) and 5 of 12 patients (41.7%) from Arm 3 (vedolizumab plus nivolumab plus ipilimumab). Among the 15 patients (68.2%) who discontinued the study, the most common reason overall was study termination by sponsor (13 patients, 86.7%), followed by withdrawal by patient (2 patients, 13.3%). Disposition by treatment arm is shown in **Figure 2**.

Arm 1 (tovorafenib plus nivolumab) was closed due to lack of enrollment. For Arm 2 (plozalizumab plus nivolumab), the sponsor stopped enrollment owing to a lack of clinical benefit, as early discontinuations were primarily due to disease progression (6 of 9 patients discontinued due to progressive disease). Enrollment in Arm 3 (vedolizumab plus nivolumab plus ipilimumab) was terminated because the prespecified stopping rule for Arm 3 was met when 2 patients (1 receiving vedolizumab at DL 1 [200 mg] and 1 at DL 2 [450 mg]) presented with grade 3 diarrhea/colitis during dose escalation.

### Study drug exposure

**Figure 3:**
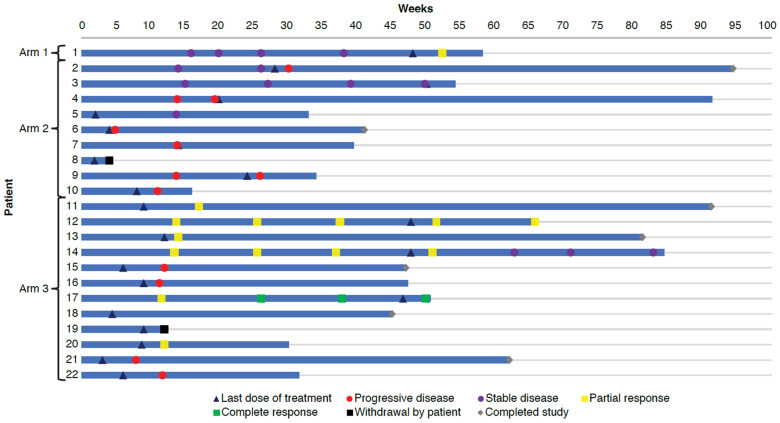
Swimmer plot.

The duration of study treatment exposure was 48.1 weeks for the 1 patient in Arm 1 (tovorafenib plus nivolumab), a median of 14.1 weeks (minimum, 2.0; maximum, 50.1) for the 9 patients in Arm 2 (plozalizumab plus nivolumab), and a median of 9.1 weeks (minimum, 3.1; maximum, 47.9) for the 12 patients in Arm 3 (vedolizumab plus nivolumab plus ipilimumab) (**Table 2**). Most patients (the single patient in Arm 1, 6 of 9 patients [66.7%] in Arm 2 and 8 of 12 patients [66.7%] in Arm 3) received study treatment for at least 8 weeks. **Figure 3** shows study drug exposure in individual patients. Cumulative dose and relative intensity of dose for each treatment are shown in **Table 2**.

### Dose-limiting toxicities

The frequency of protocol-defined DLTs was the primary endpoint of the dose-escalation phase plus the expansion Part 1. In study Arm 2, 3 patients were enrolled at the plozalizumab DL of 4 mg/kg (DL 1), and 6 patients were enrolled at the plozalizumab DL of 8 mg/kg (DL 2). In study Arm 3, 4 patients were enrolled at the vedolizumab DL of 200 mg (DL 1), and 8 patients were enrolled at the vedolizumab DL of 450 mg (DL 2).

The only DLTs in this study were reported in 1 patient from study Arm 2 who received plozalizumab 8 mg/kg plus nivolumab. The patient presented with a composite DLT of an asymptomatic grade 3 alanine aminotransferase (ALT) elevation lasting longer than 7 days accompanied by a grade 2 aspartate aminotransferase elevation; both were determined by the investigator to be related to study treatment, but neither was a serious AE. The patient underwent a liver biopsy that revealed a mild hepatic steatosis in parenchyma with mild lobular injury most consistent with a toxic or treatment-induced liver injury. The patient discontinued study treatment, received treatment with prednisone, recovered from the event, and remained in the study until it was terminated by the sponsor. No DLTs were reported in the other 2 treatment arms.

### Safety

All 22 patients experienced at least 1 TEAE. The single patient from Arm 1, 88.9% of patients from Arm 2, and all patients from Arm 3 experienced at least 1 treatment-related TEAE (**Table 3**). TEAEs of grade 3 or higher were reported in the single patient from Arm 1, 3 patients (33.3%) from Arm 2, and 10 patients (83.3%) from Arm 3. Specifically, grade 3 events were reported in 3 patients (33.3%) from Arm 2 and 8 patients (66.7%) from Arm 3; grade 4 events were reported in the single patient from Arm 1; grade 5 events, or deaths, occurring within 30 days of the last dose of study drug were reported in 2 patients (16.7%) from Arm 3.

Serious TEAEs were reported in the single patient from Arm 1, 2 patients (22.2%) from Arm 2, and 7 patients (58.3%) from Arm 3 (**Table 3**). Serious TEAEs considered to be treatment-related by investigators were reported in the one patient from Arm 1 and 3 patients (25.0%) from Arm 3 (**Table 3**). For 2 patients (22.2%) from Arm 2 and 4 patients (33.3%) from Arm 3, TEAEs led to treatment or dose discontinuation; 1 patient (11.1%) from Arm 2 and 1 patient (8.3%) from Arm 3 discontinued the study due to TEAEs. One patient who was enrolled in Arm 2 was noted to have experienced TEAEs leading to both study treatment discontinuation and study discontinuation.

There were 2 deaths, both in treatment Arm 3 (vedolizumab plus nivolumab plus ipilimumab). Deaths were reported as being caused by metastatic malignant melanoma 20 days after the last dose in one patient and acute coronary syndrome 29 days after the last dose in another patient. Neither event was determined to be related to study treatment by the investigators, and neither event was a DLT.

In Arm 1, TEAEs reported more than once included vomiting (6 events), nausea (3 events), and fatigue (2 events). In Arm 2, the most frequent TEAEs (reported by >30% of patients) included nausea (7 events in 6 patients [66.7%]); fatigue (8 events in 4 patients [44.4%]); chills and hypothyroidism (5 events in 4 patients [44.4%] each); pyrexia and diarrhea (5 events in 3 patients [33.3%] each); abdominal pain, decreased appetite and headache (4 events in 3 patients [33.3%] each); and cough (3 events in 3 patients [33.3%]). In Arm 3, the most frequent TEAEs included fatigue (12 events in 9 patients [75.0%]); nausea (10 events in 8 patients [66.7%]); pruritus (10 events in 6 patients [50.0%]); diarrhea (12 events in 5 patients [41.7%]); headache (10 events in 5 patients [41.7%]); pyrexia (9 events in 5 patients [41.7%]); abdominal pain (8 events in 4 patients [33.3%]); anemia (7 events in 4 patients [33.3%]); and adrenal insufficiency, cough and rash (4 events in 4 patients [33.3%] each).

Treatment-related TEAEs that occurred in ≥10% of patients across the 3 study arms are shown in **Table 4**. The single patient from Arm 1 (tovorafenib plus nivolumab) had 3 treatment-related grade 3 or higher TEAEs: nausea, adrenal insufficiency, and dermatitis acneiform. One patient in Arm 2 (plozalizumab plus nivolumab) had 1 treatment-related grade 3 or higher TEAE of increased ALT. Treatment-related grade 3 or higher TEAEs in Arm 3 (vedolizumab plus nivolumab plus ipilimumab) were diarrhea in 2 patients; and colitis, fatigue, increased ALT, increased international normalized ratio, decreased platelet count, hyperuricemia, hyponatremia, hypophysitis, autoimmune hepatitis, immune-mediated hepatitis, anemia, eosinophilia, and leukocytosis in 1 patient each.

### Treatment response

The single patient from Arm 1 (tovorafenib plus nivolumab) achieved partial response at the end of treatment. Of 5 patients from Arm 2 (plozalizumab plus nivolumab) who had evaluations at the end of treatment, all had progressive disease. Evaluation at the end of treatment was not conducted in 4 patients from Arm 2, of whom 1 had progressive disease, and 1 had stable disease at the last available evaluation; the other 2 patients had no evaluations during the study. Of 8 patients from Arm 3 (vedolizumab plus nivolumab plus ipilimumab) who had evaluations at the end of treatment, 1 patient had complete response, 3 patients had partial response, and 4 patients had progressive disease. Evaluation at the end of treatment was not conducted in 4 patients from Arm 3, of whom 2 had partial response at the last available evaluation and 2 had no evaluations during the study. Responses in individual patients can be seen in **Figure 3**.

## Discussion

This phase 1b clinical trial was conducted in patients with advanced melanoma who were eligible for SOC immunotherapy with a PD-1 inhibitor alone or in combination with a CTLA-4 inhibitor. SOC nivolumab was combined with either tovorafenib or plozalizumab, whereas SOC nivolumab plus ipilimumab was combined with vedolizumab. Study Arms 1 and 2 sought to advance the hypothesis that novel therapies (tovorafenib and plozalizumab) in combination with nivolumab immunotherapy could influence the tumor microenvironment, and that changes in the tumor microenvironment could predict greater antitumor efficacy with the novel combination therapy versus nivolumab alone. The hypothesis was unable to be tested for Arm 1 because of low recruitment. The hypothesis was tested for Arm 2 and did not meet the bar for efficacy to continue enrollment with this combination. Study Arm 3 sought to test the hypothesis that vedolizumab, a drug approved for the treatment of IBD, may be efficacious in preventing gastrointestinal immune-related AEs, resulting in improved clinical benefit in patients with advanced melanoma receiving combination therapy with nivolumab plus ipilimumab. Study Arm 3 was terminated early per protocol after reaching the prespecified stopping rule for diarrhea when 2 of 12 patients (16.7%) from this arm presented with grade 3 diarrhea; furthermore, diarrhea of any grade was reported in 5 of 12 patients (41.7%) from Arm 3. Although this analysis is limited by a small number of patients enrolled and shortened dosing regimens, the frequency of diarrhea/colitis events was similar to that reported for the combination of nivolumab plus ipilimumab without vedolizumab 7. The trend suggests that vedolizumab at the doses and schedule described herein has a limited effect as primary prophylaxis to prevent gastrointestinal toxicity associated with the combination of nivolumab plus ipilimumab in this patient population.

Arm 1 (tovorafenib plus nivolumab) was closed due to lack of enrollment, with just 1 patient enrolled. This arm enrolled only patients with *BRAF* V600 mutation-positive or *NRAS* mutation-positive disease previously untreated with RAF, MEK, or other inhibitors of the MAPK pathway. The availability of BRAF and MEK inhibitor combinations approved at the time this study was conducted (dabrafenib plus trametinib and vemurafenib plus cobimetinib) limited the identification of patients meeting this requirement. Subsequent results from clinical studies such as DREAMseq 36 and SECOMBIT 37, which compared immunotherapy first or targeted therapy first in patients with advanced melanoma and *BRAF* V600 mutations, were not available at the time our study was conducted, although ultimately supported the use of nivolumab plus ipilimumab as frontline therapy for this patient population. However, underlying investigator bias likely favored starting with immunotherapy. Advances in access to next-generation sequencing platforms and wider uptake of screening for melanoma with traditionally nontargetable mutations such as *NRAS* may enable more efficient identification of patients with advanced melanoma who might benefit from novel targeted therapies alone or in combination with immunotherapy in future clinical studies.

Although the combination of nivolumab plus ipilimumab has regulatory approval and is known to have greater efficacy than either agent alone, its toxicity profile may pose a barrier to its use 4-7. In a 6.5-year follow-up of the phase 3 CheckMate 067 study, treatment-related AEs were observed in 59% of patients from the nivolumab plus ipilimumab arm, versus 24% of patients from the nivolumab arm and 28% of patients from the ipilimumab arm 6. In a pooled analysis of 448 patients who received nivolumab and ipilimumab in phase 2 and 3 clinical studies, any-grade treatment-related diarrhea or colitis was reported in 44.0% and 12.7% of patients, respectively 7. Of grade 3/4 related events, diarrhea occurred in 9.8% of patients and colitis in 8.7% of patients; furthermore, diarrhea and colitis were the most frequent AEs leading to treatment discontinuation 7. A higher rate of immune-related AEs and treatment discontinuations with nivolumab plus ipilimumab combination therapy versus monotherapy limits the administration of the combination 2. In a pooled analysis of 407 patients who received nivolumab plus ipilimumab in phase 2 and 3 clinical studies, 156 patients discontinued combination treatment due to AEs; these patients received a median of 3 doses of nivolumab and ipilimumab 38.

Arm 3 of the study was designed to test whether vedolizumab could improve the gastrointestinal toxicity profile of nivolumab plus ipilimumab by acting as a primary prophylaxis against gastrointestinal immune-related AEs without loss of antitumor efficacy. Vedolizumab is indicated in adult patients for the treatment of IBD (ulcerative colitis and Crohn's disease) 29; data on vedolizumab for IBD treatment in patients with IBD and active cancer are limited and decisions on vedolizumab use in these patients are made on a case-by-case basis 39. Vedolizumab is also listed in the clinical guidelines as a treatment option for immune checkpoint inhibitor-mediated colitis following ineffective treatment with corticosteroids and/or infliximab 40, 41. However, less is known about the potential use of prophylactic vedolizumab administered concurrently with immunotherapy for immune-related AE prevention.

In our study, Arm 3 (vedolizumab plus nivolumab plus ipilimumab) was initially paused for evaluation of new AEs, reported as related by the investigators, which potentially could modify the benefit-risk profile for participating patients in this arm. Arm 3 was later terminated after meeting a prespecified stopping rule when 2 patients, 1 from each vedolizumab DL, presented with grade 3 diarrhea/colitis during dose escalation. In this study, vedolizumab dosing (4 doses of 200 mg or 450 mg: at treatment initiation and at 2, 4, and 12 weeks after the initial dose) differed from that approved for IBD treatment (4 doses of vedolizumab 300 mg for induction: at treatment initiation and at 2, 6, and 14 weeks after the initial dose, followed by 300 mg every 8 weeks thereafter for maintenance). Although the dose rationale for the current study was selected to achieve target pharmacokinetic and pharmacodynamic steady state early in the treatment, based on modelling, it may not have resulted in adequate anti-integrin α_4_β_7_ activity and expected immunomodulation, resulting in limited prophylactic effect in preventing immune checkpoint inhibitor-induced colitis when given as the studied regimen. However, retrospective data suggest that vedolizumab in combination with checkpoint inhibitors may have a role in reducing gastrointestinal toxicity in patients resuming immune checkpoint inhibitors following prior immune checkpoint inhibitor-induced colitis 32. The study results, although limited due to a small number of patients enrolled and the early termination, suggest that vedolizumab at the studied dosing regimens has a limited effect as a primary prophylaxis to prevent gastrointestinal toxicity associated with nivolumab plus ipilimumab treatment in the studied patient population. Importantly, the use of vedolizumab did not appear to attenuate the efficacy of the nivolumab plus ipilimumab combination, as 6 of the 12 treated patients in Arm 3 had a response to treatment. Concrete conclusions about the efficacy of nivolumab plus ipilimumab treatment when combined with vedolizumab cannot be made, as only the first study phase (dose escalation) was initiated before study termination. Only 12 patients were enrolled to Arm 3; most of these patients did not complete the study. The second study phase (expansion Part 1) to obtain the preliminary clinical activity in up to 15 patients and the third study phase (expansion Part 2) to evaluate the overall response rate in up to 46 patients per arm were not initiated.

Phase 1 trials are a crucial initial step in early-phase oncology drug development, facilitating evaluation of the safety and tolerability of novel investigational agents and combinations, and leading to the determination of the recommended dose in later phase studies or the same study, as was the original intent of the study reported here 42. This phase 1b study was conducted with a 3-phase design, starting with a dose-escalation safety lead-in phase with standard 3+3 escalation rules to evaluate DLTs, and together with the second phase (expansion Part 1), to determine the dose to take through to the third phase (expansion Part 2) of the study. While more contemporary Phase 1 trials often implement an adaptive statistical model such as the Bayesian Optimal Interval Design (BOIN) that allows more specificity in to identify the DLT rate, the 3+3 design has been the work horse of Phase 1 clinical development in Phase1 trials for decades and thus was employed here 43. Independent of how DLT rate was estimated in our trial, since we implemented a three phase design to investigate each combination in dose escalation, we were able to terminate each study arm early, for the reasons described previously, thereby minimizing the number of patients recruited, especially in Arms 2 and 3, when it became clear that the prespecified bar of clinical benefit for each arm was unlikely to be met. Although the early termination of this study was the obvious limitation preventing the advancement of our hypotheses, this design was also a study strength in limiting patient recruitment to arms that were unlikely to improve outcomes.

## Supplementary Material

Supplementary appendix, figure and table.

## Figures and Tables

**Table 1:** Patient demographic and baseline characteristics.

	Tovorafenib plus nivolumab (n=1)	Plozalizumab plus nivolumab (n=9)	Vedolizumab plus nivolumab plus ipilimumab (n=12)
**Age, years**			
Mean ± SD	41	69.3 ± 11.5	55.8 ± 14.9
Median (range)	41	71 (50, 78)	59 (33, 85)
**Sex, n (%)**			
Male	0	6 (66.7)	5 (41.7)
Female	1 (100)	3 (33.3)	7 (58.3)
**Race, n (%)**			
Black	0	1 (11.1)	0
White	1 (100)	8 (88.9)	11 (91.7)
Not reported	0	0	1 (8.3)
**ECOG performance status, n (%)**			
0	1 (100)	5 (55.6)	7 (58.3)
1	0	4 (44.4)	5 (41.7)
**Method of stage classification, n (%)**			
Clinical	0	1 (11.1)	2 (16.7)
Pathological	1 (100)	8 (88.9)	10 (83.3)
**Disease stage at study entry, n (%)**			
III	1 (100)	1 (11.1)	0
IV	0	8 (88.9)	12 (100)
**Sites of cancer involvement, n (%)**			
Adrena	0	0	2 (16.7)
Bone	0	2 (22.2)	1 (8.3)
Head and neck	0	0	1 (8.3)
Liver	0	3 (33.3)	4 (33.3)
Lung	0	4 (44.4)	8 (66.7)
Lymph nodes	0	2 (22.2)	7 (58.3)
Other	0	3 (33.3)	2 (16.7)
Rectum	0	1 (11.1)	0
Skin	0	2 (22.2)	3 (25.0)
Soft tissue	1 (100)	0	3 (25.0)
**Prior antineoplastic therapy for cancer under study**			
Yes	0	5 (55.6)	5 (41.7)
No	1 (100)	4 (44.4)	7 (58.3)
**Prior radiation therapy for cancer under study**			
Yes	0	3 (33.3)	0
No	1 (100)	6 (66.7)	12 (100)
**Prior surgical procedures for cancer under study**			
Yes	1 (100)	8 (88.9)	7 (58.3)
No	0	1 (11.1)	5 (41.7)

ECOG: Eastern Cooperative Oncology Group; SD: standard deviation.

**Table 2:** Study drug exposure

	Tovorafenib plus nivolumab (n=1)	Plozalizumab plus nivolumab (n=9)	Vedolizumab plus nivolumab plus ipilimumab (n=12)
**Duration of exposure, weeks**			
Mean ± SD	48.1	17.0 ± 15.7	17.6 ± 18.2
Median (range)	48.1	14.1 (2.0-50.1)	9.1 (3.1-47.9)
**Tovorafenib/plozalizumab/vedolizumab cumulative dose**			
Mean ± SD	8,600 mg	34.7 ± 16.6 mg/kg	1,150.0 ± 356.1 mg
Median (range)	8,600 mg	32.0 (16-64) mg/kg	1,350.0 (600-1,800) mg
**Tovorafenib/plozalizumab/vedolizumab relative dose intensity, %**			
Mean ± SD	82.7	94.4 ± 11.8	97.9 ± 7.2
Median (range)	82.7	100.0 (67-100)	100.0 (75-100)
**Nivolumab cumulative dose, mg/kg**			
Mean ± SD	63.0	24.7 ± 22.5	15.8 ± 23.0
Median (range)	63.0	21.0 (3-72)	3.5 (2-56)
**Nivolumab relative dose intensity, %**			
Mean ± SD	87.5	84.5 ± 22.4	87.5 ± 19.2
Median (range)	87.5	92.9 (33-100)	96.6 (43-100)
**Ipilimumab cumulative dose, mg/kg**			
Mean ± SD	—	—	9.3 ± 2.4
Median (range)	—	—	9.0 (6-12)
**Ipilimumab relative dose intensity, %**			
Mean ± SD	—	—	89.6 ± 16.7
Median (range)	—	—	100.0 (50-100)

SD: standard deviation**.**

**Table 3:** Overall safety

	Tovorafenib plus nivolumab (n=1)	Plozalizumab plus nivolumab (n=9)	Vedolizumab plus nivolumab plus ipilimumab (n=12)
All TEAEs, n (%)	1 (100)	9 (100)	12 (100)
Treatment-related TEAE, n (%)	1 (100)	8 (88.9)	12 (100)
Grade 3 or higher TEAE, n (%)	1 (100)	3 (33.3)	10 (83.3)
Grade 3 or higher treatment-related TEAE, n (%)	1 (100)	1 (11.1)	7 (58.3)
TEAE leading to drug or dose discontinuation, n (%)	0	2 (22.2)	4 (33.3)
TEAE leading to study discontinuation, n (%)	0	1 (11.1)	1 (8.3)
Serious TEAE, n (%)	1 (100)	2 (22.2)	7 (58.3)
Serious treatment-related TEAE, n (%)	1 (100)	0	3 (25.0)
Serious TEAE leading to drug discontinuation, n (%)	0	1 (11.1)	0
Deaths (within 30 days of last dose), n (%)	0	0	2 (16.7)

TEAE: treatment-emergent adverse event.

**Table 4:** Treatment-related TEAEs occurring in ≥10% of patients across all 3 treatment arms

Treatment-related TEAE, n (%)	Tovorafenib plus nivolumab (n=1)	Plozalizumab plus nivolumab (n=9)	Vedolizumab plus nivolumab plus ipilimumab (n=12)
Fatigue	1 (100)	4 (44.4)	7 (58.3)
Chills	0	3 (33.3)	0
Pyrexia	0	1 (11.1)	2 (16.7)
Influenza-like illness	0	1 (11.1)	2 (16.7)
Nausea	1 (100)	3 (33.3)	6 (50.0)
Diarrhea	0	3 (33.3)	5 (41.7)
Constipation	0	1 (11.1)	2 (16.7)
Abdominal pain	0	1 (11.1)	2 (16.7)
Dry mouth	0	1 (11.1)	2 (16.7)
Maculopapular rash	0	2 (22.2)	3 (25.0)
Rash	0	1 (11.1)	3 (25.0)
Pruritus	0	1 (11.1)	4 (33.3)
Hypothyroidism	0	4 (44.4)	3 (25.0)
Adrenal insufficiency	1 (100)	0	3 (25.0)
Hyperthyroidism	0	0	3 (25.0)
ALT increased	0	2 (22.2)	2 (16.7)
AST increased	0	2 (22.2)	1 (8.3)
Decreased appetite	0	2 (22.2)	1 (8.3)
Anemia	0	1 (11.1)	3 (25.0)

ALT: alanine aminotransferase; AST: aspartate aminotransferase; TEAE: treatment-emergent adverse event.

## Abbreviations

AE: adverse event
AJCC: American Joint Committee on Cancer
ALT: alanine aminotransferase
BRAF: B-type proto-oncogene
CCR2: cysteine-cysteine chemokine receptor type 2
CTLA-4: cytotoxic lymphocyte antigen-4
DL: dose level
DLT: dose-limiting toxicity
ECOG: Eastern Cooperative Oncology Group
IBD: inflammatory bowel disease
MAPK: mitogen-activated protein kinase
MEK: mitogen-activated extracellular signal-regulated kinase
NRAS: neuroblastoma rat sarcoma viral oncogene homolog
PD-1: programmed cell death protein-1
RAF: rapidly accelerated fibrosarcoma
RECIST: Response Evaluation Criteria in Solid Tumors
SOC: standard of care
TEAE: treatment-emergent adverse event
