# Supplementary material for: Phase 1b Study of Tovorafenib, Plozalizumab or Vedolizumab Plus Standard-of-Care Immune Checkpoint Inhibitors in Patients with Advanced Melanoma
Source: J Cancer. 2025 Aug 11;16(13):3797–809. doi: 10.7150/jca.117878 (PMC12490977; doi:10.7150/jca.117878)
Supplement: Supplementary file 1 — Supplementary appendix, figure and table. [file jcav16p3797s1.pdf]

SUPPLEMENTARY INFORMATION

Appendix 1

Study inclusion criteria

1. Male or female patients aged 18 years or older.
2. Histologically confirmed, unresectable stage III or stage IV melanoma per the American Joint Committee on Cancer (AJCC) staging system.
3. Eligible for treatment with nivolumab or nivolumab plus ipilimumab at the dose(s) and schedule(s) recommended as standard of care.
4. Eastern Cooperative Oncology Group (ECOG) performance status 0−1.
5. Adequate bone marrow reserve and renal and hepatic function within 28 days before the first dose of study drug on the basis of the following laboratory parameters: absolute neutrophil count ≥1,000/mm^3, platelet count ≥75,000/mm^3 and hemoglobin ≥8 g/dL (with or without transfusion support); total bilirubin ≤1.5 × the institutional upper limit of normal (ULN) or <3.0 × ULN in patients with Gilbert's syndrome; serum alanine aminotransferase or aspartate aminotransferase ≤3.0 × the institutional ULN (<5 × ULN if liver enzyme elevations are due to liver metastases); creatinine <1.5 × the institutional ULN or estimated creatinine clearance using the Cockcroft-Gault formula ≥50 mL/min/1.73 m^2 for patients with serum creatinine concentrations above institutional limits.
6. Suitable venous access for the collection of study-required blood sampling, including pharmacokinetic and pharmacodynamic blood samples.
7. Participation in a previous clinical study will require a washout period before first study drug administration >2 weeks or >5 times the half-life, whichever is shorter, for prior antitumor therapy (chemotherapy, targeted agents, immunotherapy, and radiotherapy) or any investigational treatment; patient has recovered from all toxic effects of previous therapy or at new baseline (patients with ongoing grade 1 events from prior therapies will be eligible); prior radiotherapy must have been completed at least 2 weeks before the first dose of study drug.
8. For Arms 1 and 2 only: Disease accessible for repeat nonsignificant risk biopsies (those occurring outside the brain, lung/mediastinum and pancreas, or obtained with endoscopic procedures extending beyond the esophagus, stomach or bowel) and willingness to undergo serial tumor biopsies.

9. Female patients who are postmenopausal for at least 1 year before the screening visit, OR are surgically sterile, OR if they are of childbearing potential, agree to practice one highly effective method of contraception and one additional effective (barrier) method of contraception at the same time, from the time of signing the informed consent form through 18 weeks after the last dose of tovorafenib, plozalizumab, or vedolizumab, or for as long as mandated by local labelling for nivolumab or ipilimumab, OR agree to practice true abstinence, when this is in line with the preferred and usual lifestyle of the patient (periodic abstinence [e.g., calendar, ovulation, symptothermal, postovulation methods], withdrawal, spermicides only, and lactational amenorrhea are not acceptable methods of contraception; female and male condoms should not be used together).
10. Male patients, even if surgically sterilized (i.e., status post vasectomy), who agree to practice effective barrier contraception during the entire study treatment period and through 18 weeks after the last dose of tovorafenib, plozalizumab, or vedolizumab, or for as long as mandated by local labelling for nivolumab or ipilimumab, OR agree to practice true abstinence, when this is in line with the preferred and usual lifestyle of the patient, during the entire study treatment period and through 18 weeks after the last dose of tovorafenib, plozalizumab, or vedolizumab, or for as long as mandated by local labelling for nivolumab and ipilimumab; periodic abstinence (e.g., calendar, ovulation, symptothermal, postovulation methods for the female partner) and withdrawal are not acceptable methods of contraception.
11. Voluntary written consent must be given before performance of any study-related procedure not part of standard medical care, with the understanding that consent may be withdrawn by the patient at any time without prejudice to future medical care.
12. For Arm 1 (tovorafenib plus nivolumab) only: B-type proto-oncogene (*BRAF*) V600 mutation−positive or neuroblastoma rat sarcoma viral oncogene homolog (*NRAS*) mutation−positive disease previously untreated with rapidly accelerated fibrosarcoma (RAF), mitogen-activated extracellular signal-regulated kinase (MEK), or other inhibitors of the mitogen-activated protein kinase (MAPK) pathway. Patients who have progressed when receiving these agents can still be enrolled in Arms 2 or 3.
13. For expansion cohorts only: measurable disease per Response Evaluation Criteria in Solid Tumors (RECIST) guidelines (version 1.1) and at least one nonsignificant risk, nontarget lesion accessible for biopsy per the guidelines above.

Study exclusion criteria

1. Female patients who are lactating and breastfeeding or have a positive serum pregnancy test during the screening period or a positive urine pregnancy test on day 1 before first dose of study drug, unless the positive pregnancy test is proven to be a false positive.
2. Any serious medical or psychiatric illness that could, in the investigator's opinion, potentially interfere with the completion of treatment according to this protocol.
3. Active brain metastases or leptomeningeal metastases. Subjects with brain metastases are eligible if these have been treated and there is no magnetic resonance imaging evidence of progression for at least 4 weeks after treatment is complete and within 28 days before first dose of study drug administration. There must also be no requirement for high doses of systemic corticosteroids that could result in immunosuppression (>10 mg per day prednisone equivalents) for at least 2 weeks before study drug administration.
4. Completed prior therapy <2 weeks before first dose and for whom adverse events related to prior therapy had not returned to baseline or improved to grade 1.
5. Active, known, or suspected autoimmune disease. Subjects with vitiligo, type I diabetes mellitus, residual hypothyroidism due to autoimmune condition only requiring hormone replacement, psoriasis not requiring systemic treatment, or conditions not expected to recur in the absence of an external trigger are permitted to enroll.
6. Condition requiring systemic treatment with either corticosteroids (>10 mg daily prednisone equivalents) or other immunosuppressive medications within 14 days of study drug administration. Inhaled or topical steroids and adrenal replacement doses >10 mg daily prednisone equivalents are permitted in the absence of active autoimmune disease.
7. History of pneumonitis requiring treatment with steroids; history of idiopathic pulmonary fibrosis (including pneumonitis), interstitial lung disease, drug-induced pneumonitis, organizing pneumonia, or evidence of active pneumonitis on screening chest computed tomography scan; history of radiation pneumonitis in the radiation field (fibrosis) is permitted.
8. Diagnosis of immunodeficiency, i.e., any identified congenital or acquired immunodeficiency (e.g., common variable immunodeficiency, human immunodeficiency virus infection, organ transplant).
9. Systemic infection requiring intravenous antibiotic therapy or other serious infection within 14 days before the first dose of study drug. Patients are specifically excluded if they have active, severe infections such as tuberculosis (screening per local practice and epidemiology), sepsis, cytomegalovirus (including cytomegalovirus colitis), listeriosis, and opportunistic infections (including *Clostridioides difficile*) until the infections are controlled.

10. Any serious or uncontrolled medical disorder that, in the opinion of the investigator, may increase the risk associated with study participation or study drug administration, impair the ability of the subject to receive protocol therapy, or interfere with the interpretation of study results.
11. Known, previously diagnosed human immunodeficiency virus infection or active hepatitis B or C. Specific screening for chronic viral illness is at the discretion of the site or local institutional review board.
12. History of known serious or severe hypersensitivity reaction to any of the study drugs or its excipients (exclusion criterion for each specific agent/arm).

Additional exclusion criteria for Arm 1 (tovorafenib plus nivolumab) only

1. Concomitant use or administration of clinically significant enzyme inducers ≤14 days before the first dose of tovorafenib.
2. Treatment with gemfibrozil (or other strong cytochrome P450 enzyme 2C8 [CYP2C8] inhibitor) within 14 days before the first dose of tovorafenib.
3. Left ventricular ejection fraction <50% as measured by echocardiogram or multiple gated acquisition scan within 4 weeks before receiving the first dose of study drug.
4. Known gastrointestinal disease or prior gastrointestinal procedure that could interfere with the oral absorption or tolerance of tovorafenib.

Additional exclusion criteria for Arm 3 (vedolizumab plus nivolumab plus ipilimumab) only

1. An abnormal objective progressive multifocal leukoencephalopathy checklist.
2. Prior exposure to rituximab, natalizumab, vedolizumab, or alemtuzumab.
3. History of any major neurological disorders, including stroke, multiple sclerosis, or neurodegenerative disease.
4. Any live vaccinations within 30 days before study drug administration except for the influenza vaccine.

Table S1. Representativeness of study participants.

**Cancer type(s)/subtype(s)/ stage(s)/condition**	Stage III or IV melanoma
**Considerations related to:**	
Sex	Overall, the melanoma incidence rate and cumulative lifetime risk of developing melanoma is higher in males versus females [1]. The melanoma incidence rate is higher in females versus males for people aged <50 years but higher in males versus females for people aged ≥50 years [2].
Age	The mean age of melanoma diagnosis is 65 years [3].
Race	Melanoma incidence is lower among American Indian and Alaska Native, Asian and Pacific Islander, and Black persons compared with non-Hispanic White persons [4].
Ethnicity	Melanoma incidence is lower in Hispanic White compared with non-Hispanic White persons [4, 5].
Geography	Melanoma incidence varies by country and region, with the highest age-standardized incidence rates reported in Australia, New Zealand, and some countries in Europe (e.g., Denmark, Germany); estimated melanoma incidence rates in Africa, Asia and South America are lower [3]. The age-standardized incidence rate of melanoma in the United States in 2020 was 16.6 cases per 100,000 persons [3].
**Other considerations**	Despite the association between increased melanoma incidence and older age, elderly patients with melanoma are underrepresented in clinical studies, with the mean age of patients in melanoma clinical studies notably lower than the mean age of individuals with melanoma diagnosis in the community [6].<br>Although melanoma has the highest incidence in non-Hispanic White persons compared with other races and ethnicities, lower melanoma-specific survival was reported in American Indian and Alaska Native, Asian and Pacific Islander, Black, and Hispanic persons compared with non-Hispanic White persons [7]. Additionally, a higher proportion of Black and Hispanic White persons versus non-Hispanic White persons are diagnosed with melanoma at regional or distant stage [5].
**Overall representativeness of the study**	Most patients enrolled in this study were White and not Hispanic or Latino, which is consistent with melanoma incidence by race and ethnicity reported in the literature.<br>Mean age in this study was lower than the expected population average, and only 50% of enrolled patients were male, although melanoma is reported to be more frequent in males than females. The small number of enrolled patients may have affected the average age and the proportion of male patients enrolled.<br>Only patients from the United States were enrolled, which may not accurately represent patients with melanoma globally.

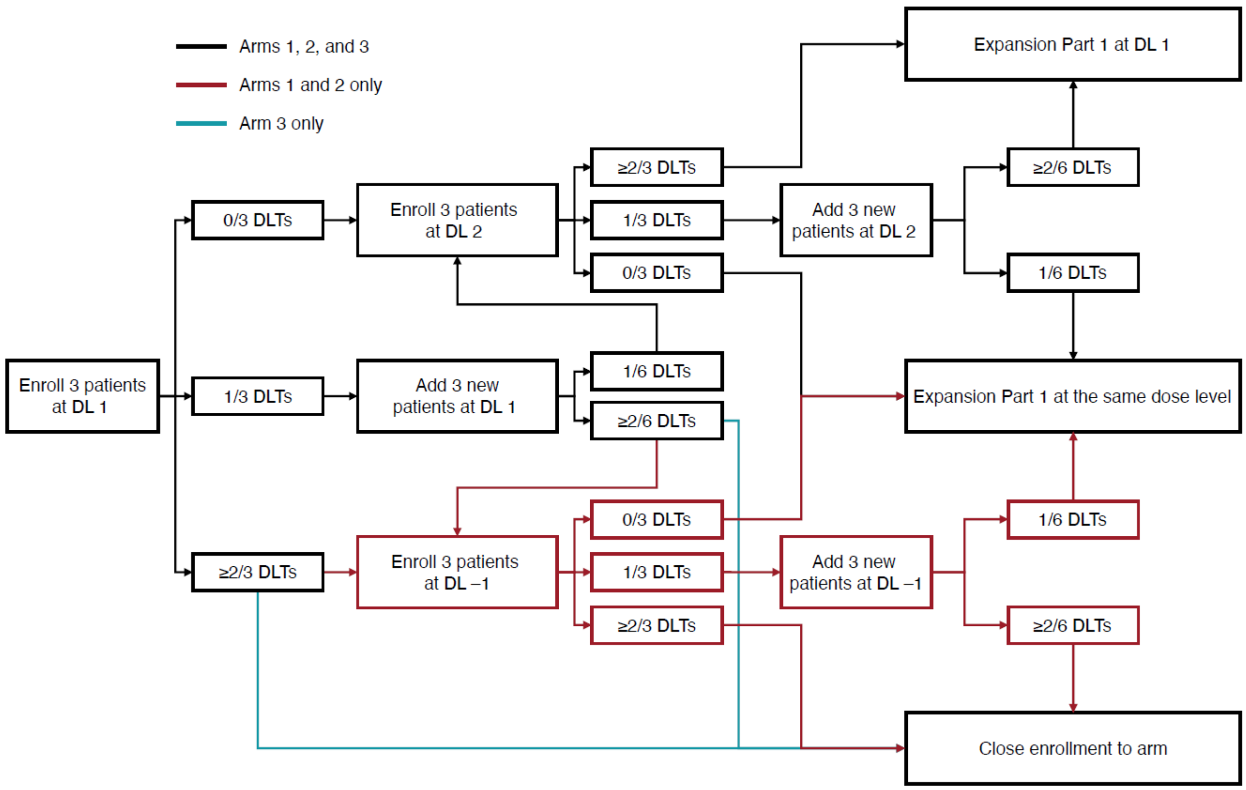

Figure S1. Dose escalation overview. Because Arm 3 (vedolizumab + nivolumab + ipilimumab) used only DL 1 and DL 2, rules leading to dose de-escalation to DL −1 in Arm 1 and Arm 2 were used to close enrollment in Arm 3 instead.

DL, dose level; DLT, dose-limiting toxicity.

Supplementary references

1. Sung H, Ferlay J, Siegel RL, Laversanne M, Soerjomataram I, Jemal A, et al. Global cancer statistics 2020: GLOBOCAN estimates of incidence and mortality worldwide for 36 cancers in 185 countries. CA Cancer J Clin. 2021; 71: 209-49.
2. Paulson KG, Gupta D, Kim TS, Veatch JR, Byrd DR, Bhatia S, et al. Age-specific incidence of melanoma in the United States. JAMA Dermatol. 2020; 156: 57-64.
3. Saginala K, Barsouk A, Aluru JS, Rawla P, Barsouk A. Epidemiology of melanoma. Med Sci (Basel). 2021; 9: 63.
4. Cronin KA, Scott S, Firth AU, Sung H, Henley SJ, Sherman RL, et al. Annual report to the nation on the status of cancer, part 1: national cancer statistics. Cancer. 2022; 128: 4251-84.
5. Hu S, Parmet Y, Allen G, Parker DF, Ma F, Rouhani P, et al. Disparity in melanoma: a trend analysis of melanoma incidence and stage at diagnosis among whites, Hispanics, and blacks in Florida. Arch Dermatol. 2009; 145: 1369-74.
6. Shah R, Patel N, Patel Y, Toscani M, Barone J, Weber PF. Age demographics of subjects enrolled in global, interventional phase 3 melanoma clinical trials. Ther Innov Regul Sci. 2022; 56: 184-90.
7. Lam M, Zhu JW, Hu A, Beecker J. Racial differences in the prognosis and survival of cutaneous melanoma from 1990 to 2020 in North America: a systematic review and meta-analysis. J Cutan Med Surg. 2022; 26: 181-8.
